# Caesarian Section

**Published:** 1899-04

**Authors:** James Byars

**Affiliations:** Local Surgeon S. P. R. R., Columbus, Texas


For Texas Medical Journal.

### Caesarian Section.—With Recovery of Mother, and Child Saved.

BY JAMES BYARS, M. D., LOCAL SURGEON S. P. R. R., COLUMBUS, TEXAS.

This is a case of a young woman, whom I had personally known for many years, and who had borne an excellent reputation up to the time of the operation.

Miss X., 26 years of age, weight in health 136 pounds, at the time of operation 116 pounds, height 5 feet 7 inches. At presentation for treatment she was sallow, anemic, and generally run down; urine highly colored and scant; specific gravity 1.025, increase of phosphates, bile and uric acid; neither albumen nor sugar; tongue was heavily coated, having a little fever in the afternoon of each day continuously for several months preceding time of operation, a typical case of chronic malarial poison. She gave the following history:

About fourteen months ago she fell half way down a flight of stairs, and was confined to her bed for several days afterwards from bruises and pains referable to the abdominal region. She felt as if "something tore loose inside of her." Had profuse sanguineous discharges for some time after the fall. Later these discharges ceased entirely, and she had not menstruated in ten and a half or eleven and a half months when I saw her. Two months after the fall she felt a lump in her side, as she described it, which for the last six weeks had grown very rapidly and was very painful at times. Had syncope, constipated bowels for several months, no evacuation unless prompted by strong purgatives, complained of pain in the right hip and thigh; appetite poor. I informed her of the necessity of an examination as to the tumor, to which she readily assented. She was placed in the dorsal decubitus, with a sheet over her, her skirts being loosened. Upon palpation, I found a tumor of the abdomen of elastic, spongy feeling, of semi-solid consistency, extending two-thirds of the distance from the pubis to the umbilicus, of ovoid shape, and occupying the middle line. I could not make out the outlines of the tumor well, on account of the distension of the intestines. The abdominal superficial veins were not enlarged, and except for a considerable increase of pigmentation of the skin there was nothing unusual. Breast and papillæ enlarged, nipples erect, and areola deeply pigmented. I attempted to examine her per vagina, and as soon as my finger entered the same, it impinged upon a round, firm body low down in the pelvis, which I took to be the virgin uterus, and before I could make out what it was she became greatly excited, cried out, and pulled away from me, so that I did not attempt to make a further examination. I inquired if she had ever experienced any movement in the tumor, and she said only a throbbing pain when she was lying on her back, and she felt no uneasiness at any other time. I concluded that I had an ovarian cyst to deal with, and the danger of its rupturing being great, on account of the rapid growth, I advised an operation, the sooner the better. With her consent I secured, in a private residence, a suitable room, and prepared for the operation, which took place on the 21st day of December, 1898.

PREPARATION FOR OPERATION.

Towels and open cheese cloth necessary were boiled in a solution of caustic soda, then thoroughly rinsed in hot water and hung up to dry. When dry they were pressed out with a hot iron, towels folded square, gauze cut in pieces half a yard in length, folded in squares and each square snugly pinned with a safety pin. Towels and gauze were then packed in a perfectly clean sheet, pinned up with safety pins, placed in a perfectly clean pan, and subjected in a bake oven to a dry heat of high temperature for two and a half hours. These were not removed from this sheet until ready for use, but when needed were placed in a hot five per cent. solution of carbolic acid. Silk ligatures were wrapped on glass rods, and the instruments were boiled for three hours, then lifted out with sterilized forceps. The silk ligatures dropped into absolute alcohol, and the instruments placed in five per cent. aqueous solution of carbolic acid, where they were allowed to remain until needed.

On the evening before the operation, the patient was given a large enema, was thoroughly scrubbed, shaved and aseptically dressed. On the morning of the operation a large enema and a vaginal wash were given. In the operation I was assisted by Dr. C. Ralph Byars, and Dr. John H. Bowers (82 years of age). Dr. Bowers gave the chloroform. We had an untrained nurse. While the patient was being placed under the influence of the anesthetic, the operating table was brought in, a plain board table, three and one-half feet high, five and one-half feet long and twenty-six inches wide, covered with a quilt and clean sheet. Patient was lifted upon the table, her legs wrapped in a clean blanket, and the clothing removed from the field of operation. Abdomen and sides were again thoroughly scrubbed with Johnson's ethereal soap and warm water, then cleansed with equal parts of ether and alcohol. The field of operation surrounded with towels wrung out of hot carbolized water; hands and arms of operator and assistants were thoroughly cleansed with soap, chlorinated lime and bicarbonate of soda, carbolized water, and lastly, with ether and alcohol in equal parts.

Incision was made along linea alba, commencing three inches above the pubis, enlarged towards umbilicus for four or five inches to easily introduce the hand. Hand introduced inside the abdominal cavity, and swept over the tumor to see as to the adhesions or dislocations of the intestines. Finding nothing abnormal, the incision was enlarged, dividing between the fingers, downwards within two inches of the pubis, and upwards, cutting around the umbilicus, till three inches above. Tumor turned out, abdominal walls tucked beneath it to prevent soiling abdominal cavity. A medium sized trocar was pushed through the wall of the tumor, to evacuate its contents, but, of course, nothing but blood escaped. Trocar was then withdrawn, and the bleeding points caught up with forceps by my son (Dr. Byars) and dropped. I then made a tranverse incision of about six inches with one stroke, cutting through the uterine wall, superior surface of placenta, and disclosing the child's buttock. Then I realized that I had operated upon a gravid uterus, instead of a tumor. I caught the uterus with my left hand low down to control the circulation, while Dr. C. R. Byars turned out the placenta and child. With my right hand I held the walls in apposition to control the hemorrhage. Child disposed of, hot gauzes were lifted into the cavity of the uterus until thorough contraction had occurred. I held the walls of the uterus in apposition while my son sutured it with eight or nine interrupted sutures, with only moderate tension. After tying the sutures, normal hemorrhage occurred only through the vagina. Uterus was then dropped back into the abdominal cavity, and one gallon of warm normal salt solution poured into same, flushed thoroughly out until the water became clear. Then one and one-half pints of the same solution was poured into the cavity and allowed to remain. In line of incision of the uterus, aristol was dusted, and also along the whole line of incision of the abdominal wound, which was closed by through-and-through interrupted silk sutures with only tension enough to coapt parts well. Towels wrung out of carbolized water were placed over the abdomen to absorb the oozing from stitch holes. After removal of towels, aristol was again dusted along the line of incision, then covered with ten per cent. iodoform gauze, cotton batting, and a towel to cover the whole. Strips of adhesive plaster, with tape attached, the plaster attached to the skin and tape tied across the dressing, which would enable me to remove the dressing by untying the tapes, and patient put to bed.

Time consumed in the operation, from first incision until the abdominal wound was closed, fifty minutes. The temperature, as results of the operation, was at no time higher than $100\frac{3}{4}°$.

Twenty-four hours after the operation I prescribed sulphate of magnesia and phosphate of soda in small doses, to be given every three hours. Although the peristaltic movements of the bowels were very active, and prevented intestinal adhesion, which I had feared, still the bowels did not move until the third day, though I had given but little morphine. This inactivity of the bowels was caused by spasm of the sphincter muscles of the anus, which was overcome by dilatation, the passing of a rectal tube, and high enema. Abdominal wound dressed the first time on the sixth day; no pus, and wound united by first intention all along the line. Wound antisepticised and sutures removed on the fourteenth day, no other dressing was required except support for abdomen. Patient was able to sit up in a chair on the sixteenth day. On the twenty-first day she had an interview with her sister, who disclosed to her the true facts, which caused great prostration, as I had never told her; neither had I permitted any one else to tell her the results of the operation. Ten hours after the interview, she had a severe rigor, and phlegmasia alba dolens in the right leg set in. The temperature rose to 102° and 104° for four or five days. Limb never swelled a great deal. Phlegmasia confined her to her bed for about fifteen days, disappearing about the twentieth day. Patient has not been discharged up to this time, but is still under treatment for intermittent malarial fever, from which she had been suffering before the operation and during recovery.

In making my diagnosis, I excluded pregnancy, as an impossibility, on account of her social standing and my personal acquaintance with her. The elastic, spongy feeling of what I thought to be a tumor, was, after the operation, explained by the large size of the placenta, which occupied the whole of the anterior and superior surface of the uterus, the child lying low down in its posterior portion.

In conclusion, I beg to state that neither Dr. J. H. Bowers nor Dr. C. R. Byars, examined the patient, and did not influence me in making my diagnosis. Child, at the time of delivery, about seven and one-half or eight months, and is now enjoying perfect health.

March 16, 1899.

